# Poly(L-lactic acid) Reinforced with Hydroxyapatite and Tungsten Disulfide Nanotubes

**DOI:** 10.3390/polym13213851

**Published:** 2021-11-08

**Authors:** Ofek Golan, Hila Shalom, Ifat Kaplan-Ashiri, Sidney R. Cohen, Yishay Feldman, Iddo Pinkas, Rakefet Ofek Almog, Alla Zak, Reshef Tenne

**Affiliations:** 1 Department of Materials Engineering, Azrieli College of Engineering, Jerusalem 9103501, Israel; ofekgo1994@gmail.com (O.G.); rakefetof@jce.ac.il (R.O.A.); 2 Department of Molecular Chemistry and Materials Science, Weizmann Institute, Rehovot 76100, Israel; hilasha4@gmail.com; 3 Chemical Research Support Department, Weizmann Institute, Rehovot 76100, Israel; ifat.kaplan-ashiri@weizmann.ac.il (I.K.-A.); Sidney.Cohen@weizmann.ac.il (S.R.C.); Isai.Feldman@weizmann.ac.il (Y.F.); iddo.pinkas@weizmann.ac.il (I.P.); 4 Department of Sciences, Holon Institute of Technology, Holon 58102, Israel; alzak@hit.ac.il

**Keywords:** PLLA, hydroxyapatite, WS_2_ nanotubes, biodegradable polymers, mechanical properties

## Abstract

Poly(L-lactic acid) (PLLA) is a biocompatible, biodegradable, and semi-crystalline polymer with numerous applications including food packaging, medical implants, stents, tissue engineering scaffolds, etc. Hydroxyapatite (HA) is the major component of natural bone. Conceptually, combining PLLA and HA could produce a bioceramic suitable for implants and bone repair. However, this nanocomposite suffers from poor mechanical behavior under tensile strain. In this study, films of PLLA and HA were prepared with small amounts of nontoxic WS_2_ nanotubes (INT-WS_2_). The structural aspects of the films were investigated via electron microscopy, X-ray diffraction, Raman microscopy, and infrared absorption spectroscopy. The mechanical properties were evaluated via tensile measurements, micro-hardness tests, and nanoindentation. The thermal properties were investigated via differential scanning calorimetry. The composite films exhibited improved mechanical and thermal properties compared to the films prepared from the PLLA and HA alone, which is advantageous for medical applications.

## 1. Introduction

Poly(L-lactic acid) (PLLA) is a biocompatible, degradable, and semi-crystalline polymer. It is one of the most investigated polymers for biodegradable/biocompatible applications including food packaging [1,2,3], medical implants [4,5,6,7], tissue engineering scaffolds [8,9], and many more [10]. PLLA can be processed by various techniques, including extrusion, solvent casting, 3D printing, electrospinning, etc. [11]. Hydroxyapatite (Ca_10_(PO_4_)_6_(OH))_2_ is the major component of natural bone [12]. Hydroxyapatite (HA) has many stoichiometric phases, called calcium phosphate phases, with Ca/P ratios varying between 1.67 and 1.5 [13]. All the calcium phosphate nonstoichiometric phases are biocompatible, induce bone repair by osteoconductivity and osteoinductivity, and, furthermore, exhibit good mechanical properties. Despite these attributes, their major disadvantage for medical applications is their brittleness [14]. Incorporated hydroxyapatite in the PLLA matrix can improve the flexibility of the bioceramic HA and consequently, produce biodegradable ceramic-polymer composites, which can be an alternative to the traditional materials used for implants or bone repair and for tissue engineering. However, both PLLA and HA and their composites suffer from low toughness, which limits their application in the human body [15,16]. Reinforcing the PLLA/HA composite with nanotubes of WS_2_ (INT-WS_2_) can remedy this disadvantage [6,17,18]. The INT-WS_2_ are multiwall nanostructures 1–20 μm long with diameters of 30–150 nm (aspect ratios of 50–100 and even larger). They are nontoxic [19,20,21,22] with very good mechanical properties (Young’s modulus 150–170 GPa, bending modulus of 217 GPa, tensile strength between 10 and 22 GPa, and strain ε > 10%) [23]. Thus, by classical polymer reinforcement concepts, they would be expected to enhance the mechanical behavior of polymer composites under optimal conditions. However, the interface between polymer and filler is in fact a critical factor in determining the ultimate composite strength [24]. This is particularly relevant for nanocomposites. For carbon nanotubes, the effects of aspect ratio and interfacial strength have been thoroughly studied [25]. Such bonding is a mixed bag—strong bonding leads to strong, but brittle composites, while weak bonds can produce a weaker but tougher composite [17]. Nonetheless, it has been shown that structurally-modified fibers can enhance both strength and toughness [26].

Interfacial properties can be enhanced by appropriate chemical modifications: INT-WS_2_ can also be readily functionalized [27,28] and dispersed in organic solvents, polymers, epoxy resins, etc. [29,30,31,32]. HA does not disperse well in the PLLA matrix and tends to agglomerate as secondary particles a few micrometers in size. This is because HA is hydrophilic, while the organic solvents used to dissolve the polymers are mostly hydrophobic [33]. However, oleic acid (OA) is an amphiphilic surfactant, used to mediate the interaction between the HA (hydrophilic ceramic) and a hydrophobic polymer, like PLLA [34]. Therefore, OA induces a homogeneous dispersion of the HA in the PLLA matrix. Previous studies conducted on PLLA/HA/INT-WS_2_ nanocomposites indicated their potential for medical applications [35,36,37,38]. 

This report focuses on the preparation and characterization of PLLA/HA bioceramic films reinforced by small amounts of INT-WS_2_. The nanocomposite was prepared via solvent casting using OA as a dispersant. The nanocomposite morphology was investigated by high-resolution scanning electron microscopy (HR-SEM). The structural aspects were investigated by X-ray diffraction (XRD), micro-Raman spectroscopy, and Fourier transform infrared spectroscopy (FTIR). Their mechanical properties were determined by tensile testing, micro-hardness, nanomechanical testing, and their thermal properties by differential scanning calorimetry (DSC).

## 2. Materials and Methods

### 2.1. Materials

Poly L-lactic acid (PLLA) with an inherent viscosity midpoint of 2.4 dL/g was purchased from Corbion (Gorinchem, The Netherlands). Oleic acid (OA, ≥99%) and Hydroxyapatite (HA, nanopowder, <200 nm particle size (BET), ≥97%, synthetic) were purchased from Sigma Aldrich Chemical Company (St. Louis, MO, USA). 

**Figure 1:**
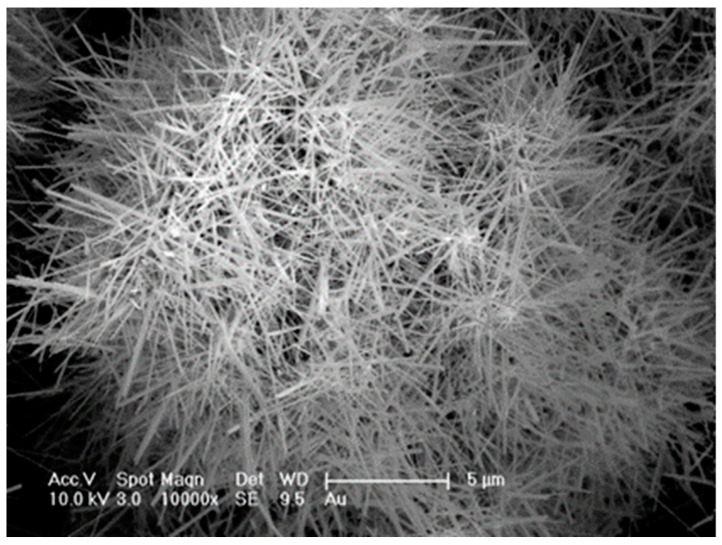
SEM view of an assortment of WS_2_ nanotubes.

INT-WS_2_ with diameters between 30 and 150 nm and lengths between 1 and 20 micrometers were synthesized using a published procedure [39]. Briefly, the precursor nanoparticles of tungsten trioxide, grow into high aspect ratio tungsten suboxide nanowhiskers under a mild reducing atmosphere at 840 °C. Subsequent sulfurization of the nanowhiskers results in hollow WS_2_ nanotubes. A representative SEM image of the typical INT-WS_2_ is shown in Figure 1.

The PLLA/HA/INT films were prepared by the solvent casting method according to the following procedure.

- PLLA neat films: 0.75 g of PLLA powder was dissolved in 20 mL chloroform and mechanically mixed. Subsequently, the solution was poured onto a Teflon plate for drying in the hood with an aluminum foil cover punctuated with 10 holes.
- PLLA films with 40 wt% hydroxyapatite: 0.75 g of PLLA powder was dissolved in 15 mL chloroform; 300 mg of hydroxyapatite nanoparticles were mixed with 5 mL chloroform and 150 µL oleic acid for 30 min. The two solutions were mixed together using a magnetic stirrer for 5 min before pouring onto a Teflon plate and were then dried in the hood using an aluminum foil cover punctuated with 10 holes.
- PLLA films with 0.5 wt% INT-WS_2_: first, 3.8 mg INT-WS_2_ powder was dispersed in 5 mL ethanol for 3 min and vacuum annealed for 1.5 h at 80 °C. Next, 0.75 g of PLLA powder was dissolved in 15 mL chloroform and mechanically mixed for 5 h; then the annealed INT-WS_2_ were dispersed in 5 mL chloroform for 3 min. Finally, the two solutions were mixed together using a magnetic stirrer for 5 min before pouring onto a Teflon plate for drying in the hood with an aluminum foil cover punctuated with 10 holes.
- PLLA films with 40 wt% hydroxyapatite and 0.25, 0.5, and 0.75 wt% INT-WS_2_: first, 1.9, 3.8, or 5.6 mg of INT-WS_2_ powder were dispersed in 5 mL ethanol for 3 min and vacuum annealed for 1.5 h at 80 °C. Next, 0.75 g PLLA powder was dissolved in 10 mL chloroform, while 300 mg of hydroxyapatite nanoparticles were dispersed in 5 mL chloroform and 150 µL oleic acid for 30 min. Afterward, the annealed INT-WS_2_ were dispersed in 5 mL chloroform for 3 min. Finally, the three solutions were mixed together using a magnetic stirrer for 5 min before pouring onto a Teflon plate for drying in the hood with an aluminum foil cover punctuated with 10 holes.

The dried samples were vacuum annealed for 5 days at 45 °C [36]. The thickness of the films was determined by caliper and was, on average, 80 μm for pure PLLA; 113 μm for PLLA+HA; 93 μm for PLLA+HA+INT. The densification of the films upon the addition of the nanotubes is attributed to the alignment of the polymer molecules along the nanotube surface. The texturing of the polymer molecules is indirect evidence of the non-specific but nevertheless strong nanotube–polymer interfacial interaction and explains the mechanical reinforcement of the PLLA by the INT (see below). 

### 2.2. Characterization Techniques

#### 2.2.1. X-ray Diffraction

X-ray diffraction (XRD) was performed for various nanocomposite films in reflection geometry using Rigaku (Tokyo, Japan) theta-theta diffractometers: an Ultima III equipped with a sealed cooper anode tube operating at 40 kV/40 mA and a TTRAX III equipped with a rotating copper anode X-ray tube operating at 50 kV/200 mA. A scintillation detector was aligned at the diffracted beam after a bent Graphite monochromator, which was used for X-ray cleaning, effectively removing K_β_. 2θ/θ scans were carried out at specular conditions in Bragg–Brentano mode with variable slits with a step size of 0.025° and a scan speed of 0.5 degrees per minute. Quantitative phase analysis, degree of crystallinity, and crystallite size (coherent scattering length) estimations were made using the Pawley-based [40] Whole Pattern Fitting (WPF) module in the Jade Pro software (Materials Data, Inc., Livermore, CA, USA) and PDF-4+ 2020 database (ICDD). According to the Pawley method, the structural data for each component of the nanocomposite film is fitted to the measured XRD pattern by non-linear least-squares optimization at each diffraction point. The modeling parameters include instrumental zero error, the intensity of each reflection with indices (*hkl*), profile parameters, and lattice constants. The crystallite sizes were also estimated according to the Scherrer formula.

#### 2.2.2. High-Resolution Scanning Electron Microscopy (HR-SEM)

For HR-SEM, cross-sections of the samples were prepared by breaking the film, which was first immersed in liquid nitrogen for ten minutes for hardening. The composite samples were mounted on aluminum stubs using two-sided carbon tape. Scanning electron microscope (SEM) images were acquired using the Ultra 55 (Zeiss, Oberkochen, Germany). at 8 kV and 30 μm aperture, in both secondary electron (SE) and backscattering electron (BSE) modes. SEM imaging was followed by the acquisition of energy-dispersive X-ray spectroscopy (EDS) hypermaps with a four-quadrant retractable detector mounted above the sample (QUANTAX FlatQUAD EDS, Bruker, Billerica, MA, USA). The normalized X-ray intensity maps of the following element lines were produced from the EDS data: C-Kα, O-Kα, S-Kα, P-Kα, Ca-Kα, and W-M. In order to avoid sample charging that could alter the effective potential on the sample surface and therefore influence the EDS measurements, the samples were coated with a conductive carbon film, 5 nm thick using the CCU-010 HV coater (Safematic, Zizers, Switzerland), prior to the EDS analysis. The EDS signals were acquired at 8 kV. This energy was set according to the highest-energy X-ray line that was measured, in this case, Ca-Kα (3.69 keV). For successful EDS measurements, the acceleration voltage should be set to 1.5–3 times the highest measured-energy line.

#### 2.2.3. Tensile Testing

The mechanical properties of the films under tension were measured by performing tensile tests, using an Instron-5965 (Instron, Norwood, MA, USA) equipped with a 5 kN load cell at room temperature and a stretching speed of 1 mm/min. The samples were cut into strips 5 mm wide and 50 mm long. The gauge length of the tested strip was 30 mm. Three specimens were tested for each type of sample, and the results were given as average values and standard deviation. The load and displacement were recorded by dedicated software provided by the manufacturer (Bluehill3, Norwood, MA, USA). The significance of the differences between samples was evaluated using a 2-tailed *T*-test.

#### 2.2.4. Micro-Hardness Test

Micro Vickers hardness test was performed using HMV-G21 ST (Shimadzu, Tokyo, Japan). A load of HV 0.025 (0.2452 N) was applied to the sample and held for 10 sbefore release. Prior to the measurement, each sample was rubbed 5 times on a silicon-carbide (SiC) paper (1200 grit). Five indentation tests were performed for each type of sample, and the results were given as average values and standard deviation.

#### 2.2.5. Nanomechanical Testing

Nanoindentation was performed on a XP Nanoindenter (KLA-Tencor, Milpitas, CA, USA), using a 10 µm diameter spherical diamond indenter tip. The area function of this probe was calibrated on a sample of poly (methyl methacrylate), with Young’s modulus of 2.9 GPa. The samples (approximately 100 micrometers thick) were attached to the stub with a very thin layer of epoxy. Indentations were performed to a depth of 1200 nm at a constant strain rate of 0.1 s^−1^, using continuous stiffness measurement (CSM) [41]. In this method, a small (2 nm) oscillation is superimposed on the loading ramp, which allows continuous measurement of Oliver and Pharr modulus and hardness with depth [42]. The reported measurements represent average CSM values between 400 and 800 nm depth, over which the traces have levelled out to a constant value. Sets of 8 indentations were made at 2–3 different positions on the sample, and indentations for which the curve did not level out by 400 nm depth were discarded.

#### 2.2.6. Differential Scanning Calorimeter (DSC)

DSC Q200 (TA Instruments, New Castle, DE, USA) was used for the analysis of the thermal properties of the samples and their degree of crystallinity. The samples were placed in an aluminum pan and heated from 30 °C to 200 °C; the temperature 200 °C was held for 3 min then lowered back to 30 °C at a rate of 10 °C/min and held for 3 min. This cycle was performed in order to erase the thermal history. In the second series of measurements, the temperature was raised to 200 °C, held for 3 min and lowered back to 30 °C. Here the temperature scan was performed at a rate of 10 °C/min and under a 50 mL/min nitrogen flow rate. From the midpoint of the (heating scan) thermograms, the glass transition (*T_g_*), cold crystallization (*T_cc_*), and melting (*T_m_*) temperatures were determined. The crystallization temperature (*T_c_*) was determined from the cooling scan. The degree of crystallinity was calculated from the DSC curves in two ways:

(1) $$X_{c}=\frac{\left(∆H_{m}-∆H_{cc}\right)}{∆H_{m}^{{}^{\circ}}}\times 100\%$$

for heating [43,44], and

(2) $$\left(1-\lambda \right)=\frac{∆H_{c}}{∆H_{m}^{{}^{\circ}}}$$

for cooling [45].

Δ*H_m_*, Δ*H_cc_* (heating), and Δ*H_c_* (cooling) are the melting enthalpy, cold crystallization enthalpy, and crystallization enthalpy (J/g), respectively; $∆H_{m}^{{}^{\circ}}$ is the heat of fusion for completely crystallized PLA (93 J/g).

#### 2.2.7. Micro-Raman Spectroscopy

Raman spectroscopy was performed using a Horiba-Jobin Yivon (Lille, France) LabRAM HR Evolution micro-Raman set-up with a 532 nm wavelength, solid-state laser. Both spectral measurements of the samples and the Raman mapping of PLLA film with 40 wt% HA and 0.5 wt% INT-WS_2_ were carried out using 600 grooves/mm grating, spectral range between 70 and 1700 cm^−1^, with 30 s for each spectrum. The reported spectra represent the average of two measurements. The Raman spectra were obtained over all composites of PLLA and HA and INT-WS_2_ film, while the mapping was performed on an area of 10 × 10 µm^2^ of the PLLA film with 40 wt% HA and 0.5 wt% INT-WS_2_.

#### 2.2.8. Fourier Transform Infrared Spectroscopy (FTIR)

Attenuated total reflectance Fourier transform infrared spectroscopy (ATR-FTIR) was performed using Alpha-T (Bruker, Billerica, MA, USA) in the range of 375 cm^−1^ to 4000 cm^−1^.

## 3. Results and Discussion

### 3.1. X-ray Diffraction

**Figure 2:**
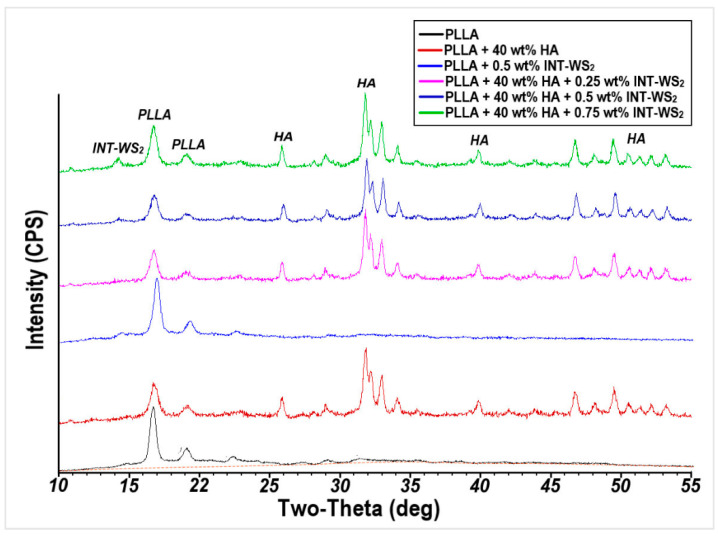
XRD patterns of PLLA film and HA/INT-WS_2_/PLLA nanocomposites.

A comparison between the XRD patterns of the nano-composite films and HA powder is shown in Figure 2. The XRD pattern of the composite PLLA and HA and INT-WS_2_ film contains peaks of the different components, which indicates that the composition and structure of PLLA and HA and nanotubes are not affected by the fabrication process of the film.

Using the Pawley-based WPF analysis, the degree of crystallinity of the samples was calculated by comparing the total area under all the crystal peaks and the area under the amorphous halo. The results are presented in Table 1. The degree of crystallinity of the PLLA film is calculated to be 32.8%. After adding 0.5 wt% of INT-WS_2_ the degree of crystallinity is nearly unchanged at 33.2%. However, after adding HA nanoparticles to the PLLA film, the degree of crystallinity increased significantly to 37.1%, rising slightly more after the addition of INT-WS_2_ at the different wt% values. 

The average crystallite size of the different compositions was estimated using the Williamson–Hall approach from the XRD peak widths fitted in the Pawley-based WPF analysis and the Scherrer equation using the main peak of the PLLA at 16.5° and is also reported in Table 1. The largest crystallite size (171 Å) occurs for the neat PLLA film. As expected, the foreign ingredients (HA and INT) serve as crystallization nuclei for the PLLA and reduce its average crystallite size.

### 3.2. High-Resolution Scanning Electron Microscopy (HR-SEM)

**Figure 3:**
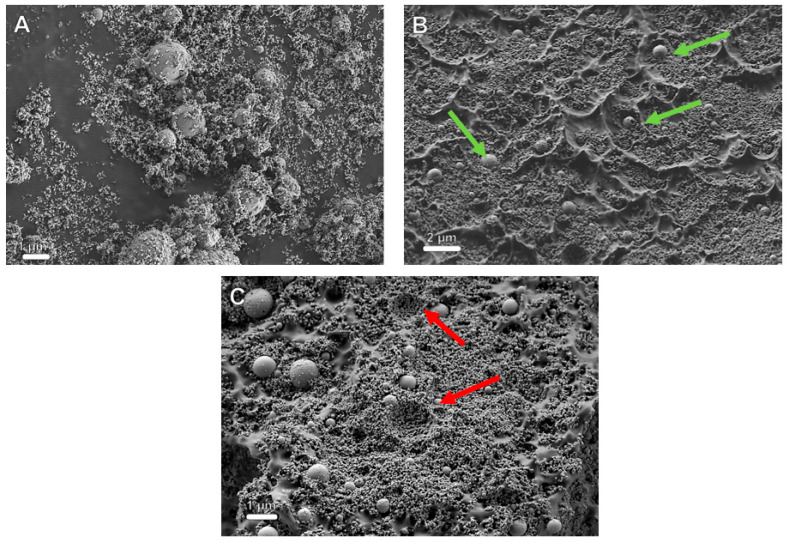
HR-SEM images (secondary electrons—SE mode) of pure HA powder (**A**) and PLLA film reinforced with 40 wt% HA observed in the SE mode at two magnifications, scale bars are 2 µm (**B**) and 1 µm (**C**). Green arrows point to the spherical agglomerates of HA nanoparticles. Red arrows point to pits formed by the pullout of the HA agglomerates during film breakage.

Figure 3 shows the SEM images of HA powder (Figure 3A), and a cross-section of PLLA with 40 wt% HA film in secondary electron (SE) mode (Figure 3B,C). Visibly (Figure 3A), the HA particles constitute a bimodal size distribution made of micron-size agglomerates and a majority phase of well-dispersed HA nanoparticles (<50 nm). The surface of the large agglomerates is decorated with small HA NPs. However, the same agglomerates appear to have a smooth and uniform surface, i.e., free of the decorating HA NPs after being incorporated into the polymer (Figure 3B—marked with green arrows). To further understand this effect, the HA phase was washed in an ultrasonic bath with the polymer-free solvent (chloroform) containing OA. The surface of the large spherical HA agglomerates was smooth and free of the HA NP decoration after this washing procedure. Therefore, the solvent treatment appears to be responsible for the “cleaning” of the HA spherical agglomerates. These smooth spherical agglomerates of HA are likely to impair the mechanical properties of the film.

Figure 3B,C show that the HA nanoparticles (NPs) were well dispersed in the polymer matrix. i.e., no phase separation or excessive additional agglomeration was observed, which was not the case in the absence of OA. Furthermore, Figure 3C shows that the HA agglomerates (>0.5 μm) were not damaged during the film breaking process, but were uprooted as a whole from the polymer matrix surface. Furthermore, the hemispherical depressions in Figure 3C (red arrows) appear to be remnants of entire HA agglomerates, which were uprooted from the polymer matrix during fracture, possibly being stuck to the other surface of the broken contact. Consequently, one can conclude that the strain was not well transferred to these agglomerates during fracture, and hence they adversely affected the mechanical strength of the film.

**Figure 4:**
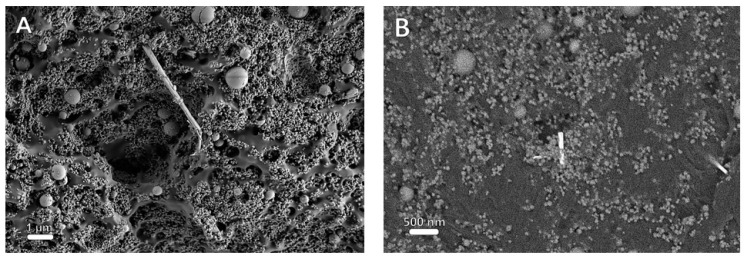
HR-SEM images of PLLA film reinforced with 40 wt% HA and 0.75 wt% INT-WS_2_ film in SE mode (**A**), scale bar is 1 µmm (**B**) BSE mode, scale bar is 500 nm.

HR-SEM images of the cross-section of PLLA reinforced with 40 wt% HA and 0.75 wt% INT-WS_2_ are presented in Figure 4. It can be seen in Figure 4A that there is no phase separation and consequently the nanotubes PLLA and the HA NPs are compatible and form a uniformly mixed matrix. The nanotubes protrude from the broken surface, which indicates that they carry some of the applied stress transferred to them from the matrix. In addition, BSE analysis shows that the INT-WS_2_, which are shown as bright stripes due to their relatively high atomic number, are fully dispersed in the PLLA matrix.

Visibly, the nanotubes protrude from the PLLA matrix, suggesting that they reinforce the polymer via bridging and pullout mechanisms [46,47].

**Figure 5:**
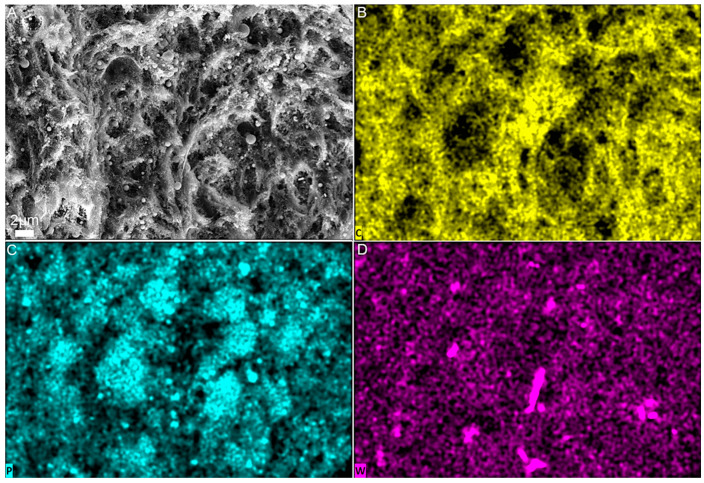
HR-SEM image of PLLA film reinforced with 40 wt% HA and 0.75 wt% INT-WS_2_ in SE mode (**A**) and EDS elemental mapping of the same area: carbon (**B**), phosphorus (**C**), and tungsten (**D**).

EDS elemental mappings of the PLLA film with 40 wt% HA and 0.75 wt% INT-WS_2_ are presented in Figure 5. The carbon mapping displayed in Figure 5B shows that the strong carbon signal is evenly distributed throughout the film. This observation reflects the fact that the matrix of the material is PLLA whose chemical composition is mostly carbon. Figure 5C presents the phosphorus mapping, which is a major component of HA. It can be seen that the HA NPs are well dispersed throughout the film. The bimodal (size) distribution with distinct micron and submicron-sized spherical agglomerates and evenly distributed HA nanoparticles is clearly discernable. The INT-WS_2_ distribution is represented by the tungsten mapping in Figure 5D, which shows that the nanotubes are well dispersed in the PLLA matrix.

### 3.3. Tensile Test

**Figure 6:**
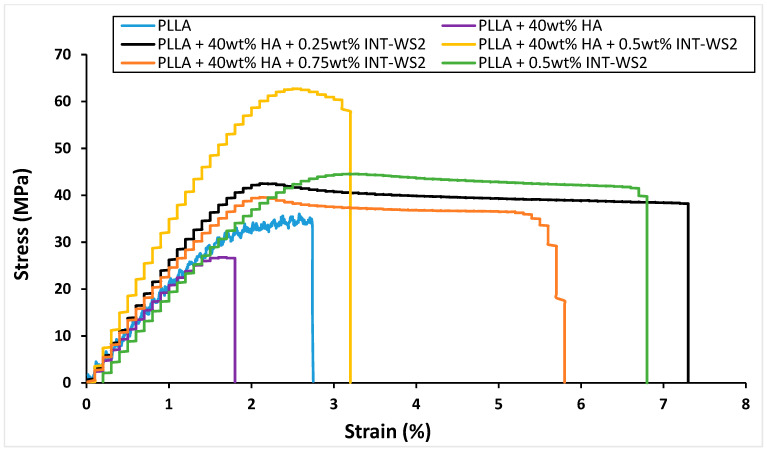
Stress–strain curves of PLLA film and its HA/INT-WS_2_/PLLA nanocomposites.

The mechanical properties derived from the stress–strain curves of the films are displayed in Figure 6 and are also presented in Table 2.

The Young’s modulus of PLLA film with 40 wt% HA (2.4 GPa) increased 1.5 times compared to the neat PLLA film (1.55 GPa), while the yield strength (26.7 MPa) and strain at failure (2.1%) of PLLA film with 40 wt% HA decreased to 0.85 and 0.75 of their values, respectively. Therefore, the toughness (area under the curve) of PLLA film with 40 wt% HA (0.3 MPa) was reduced by half compared to that of neat PLLA film (0.6 MPa). This is not surprising, since the HA is an oxide with a small strain to failure. Also, the binding of the HA to the PLLA is not chemical in nature and is rather weak (mostly van der Waals and polar interactions). These two factors adversely affect the fracture toughness of the composite. However, the indentation hardness and modulus of the PLLA+HA composite are appreciably higher than that of pure PLLA (see below). Obviously, the most rational way to mediate between the HA and the PLLA phases and increase the mechanical properties of the nanocomposite would be through surface functionalization [48]. The surface functionalization must have chemical versatility and biocompatibility in order to permit the three constituents (PLLA, HA, and INT-WS_2_) to optimally interact with each other and exhibit no biotoxicity effects.

The Young’s modulus and yield strength of PLLA film with 0.5 wt% INT-WS_2_ (2.25 GPa and 44.6 MPa, respectively) increased 1.45 times compared to the neat PLLA film, while the strain at failure of the film with 0.5 wt% INT-WS_2_ (6.8%) increased 2.5 times. Therefore, the toughness (area under the curve) of the PLLA film with 0.5 wt% INT-WS_2_ (2.4 MPa) increased significantly by four times compared to the toughness of the neat PLLA film [36].

The Young’s modulus of the PLLA film with 40 wt% HA and 0.5 wt% of INT-WS_2_ (3.8 GPa) increased up to 1.7 times compared to the PLLA film with 40 wt% HA and the PLLA film with 0.5 wt% INT-WS_2_. The yield strength of the PLLA film with 40 wt% HA and 0.5 wt% of INT-WS_2_ (62.7 MPa) increased by 2.35 and 1.4 times compared to the PLLA film with 40 wt% HA and the PLLA film with 0.5 wt% INT-WS_2_. The strain at failure of the PLLA film with 40 wt% HA and 0.5 wt% of INT-WS_2_ (3.2%) increased 1.5 times compared to the PLLA film with 40 wt% HA. However, the PLLA film with HA and 0.5 wt% INT-WS_2_ had strain at failure only half the value of the PLLA film and 0.5 wt% of INT-WS_2_. Therefore, the toughness of the PLLA film with 40 wt% HA and 0.5 wt% INT-WS_2_ (1.4 MPa) increased significantly by 4.7 times compared to the PLLA film with 40 wt% HA and decreased to 0.6 times the value of the PLLA film with 0.5 wt% INT-WS_2_.

### 3.4. Micro-Hardness Test

**Figure 7:**
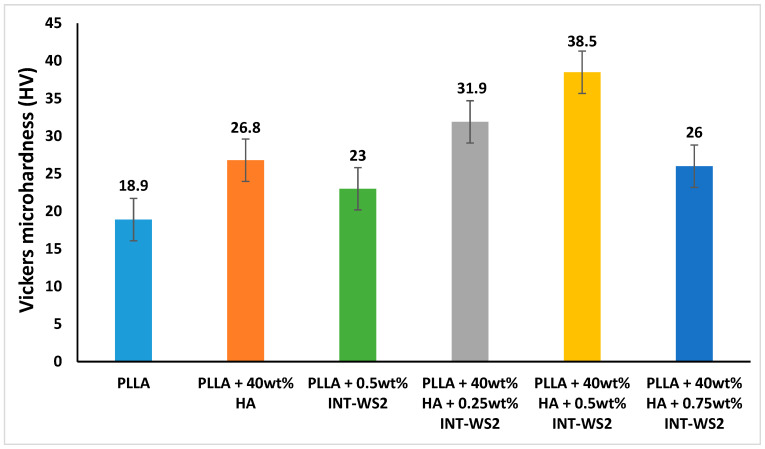
Vickers micro-hardness test of PLLA film and HA/INT-WS_2_/PLLA nanocomposites.

Figure 7 shows the results of the micro-hardness test of PLLA film and the PLLA/HA/INT-WS_2_ nanocomposites.

The addition of HA nanoparticles to the PLLA film increased the hardness value (26.8 HV) by 1.4 times compared to the hardness value of the neat PLLA film (18.9 HV). In addition, the hardness of the PLLA film with 0.5 wt% INT-WS_2_ (23 HV) increased 1.2 times compared to the hardness of the neat PLLA film, with a significance level of *p* = 0.1 for a two-tailed T-test.

A more significant increase in hardness was achieved with the combination of HA and INT-WS_2_ in PLLA. The optimum hardness value was obtained for the films containing PLLA with 40 wt% HA NPs and 0.5 wt% INT-WS_2_ with 38.5 HV, a value that is two times higher than the hardness of the pure PLLA film. It can be deduced that a small number of nanotubes added to the matrix can bridge the gap between the HA nanoparticles creating a uniform network of hardening material [49,50]. Beyond the optimal concentration of 0.5 wt%, the nanotubes have a deleterious effect on the hardness of the nanocomposite, likely due to agglomeration [51].

### 3.5. Nanomechanical Testing (Nanoindentation Tests)

While micro-hardness measurements provide an average hardness value, the small domain size in the present nanocomposite calls for a more local measurement, which serves to reveal inhomogeneities in the film. The results from the nanoindentation analysis are presented in Table 3 and are consistent with the Vickers micro-hardness tests reported above (Section 3.4).

The addition of 0.5 wt% INT-WS_2_ to the PLLA film caused almost no change in the Young’s modulus (3.4 GPa) and hardness (0.18 GPa) values compared to the parameters of the neat PLLA film with Young’s modulus of 3.3 GPa and hardness of 0.16 GPa. However, the Young’s modulus and hardness of the PLLA film with 40 wt% HA (4.9 GPa, 0.24 GPa) each increased by 1.5 times compared to the Young’s modulus and hardness of the neat PLLA film. The addition of a small number of nanotubes to the PLLA film with HA increased the Young’s modulus and hardness significantly with the optimum being at the lowest concentration measured—0.25 wt% INT-WS_2_. The Young’s modulus and hardness of the PLLA film with 40 wt% HA and 0.25 wt% INT-WS_2_ (5.6 GPa, 0.36 GPa) were significantly larger than that of the neat PLLA film, increasing by 1.7 and 2.25 times, respectively. These values were also higher than those for the PLLA+HA. Notwithstanding the large fraction of HA in the film (40 wt%), the hardness values measured here are more than an order of magnitude lower than those reported for a pure HA single crystal [52]. We ascribe the relatively low hardness to the presence of HA NPs agglomerates, which degrade the mechanical properties of the material as suggested above (Section 3.2), as well as the weak links between the HA and the PLLA. Should the HA, PLLA, and the INT be intimately and strongly bound together via surface functionalization, the picture would probably be different and the overall mechanical properties of the nanocomposite would be much improved. 

Larger statistical variations for some of the composite samples are consistent with local inhomogeneities in the nanoparticles’ distribution, as is supported by the EDS measurements and mapping, and the Raman studies (below). Nanoindentation results show relative uncertainties an order of magnitude higher in comparison with the micro-hardness data. This can be attributed to the scale of the inhomogeneities within the sample: EDS mappings in Figure 5 show HA “pockets” of several μm extent, and WS_2_ inhomogeneities on a smaller scale. The area of the microindentation imprint varies between 1000 and 2500 μm^2^ (axial length of 30–50 μm) whereas for the nanoindentations the relevant indentation size is 4–5 μm, which is on the scale of the HA pockets, and INT length.

### 3.6. Thermal Properties of PLLA Film and HA/INT-WS_2_/PLLA Nanocomposites by DSC

**Figure 8:**
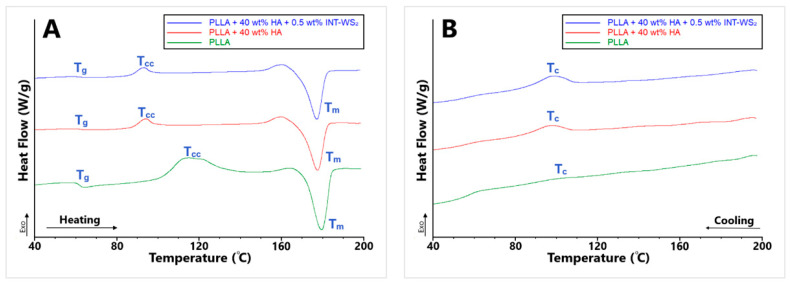
DSC thermograms of heating (**A**) and cooling (**B**) of PLLA film and HA/INT-WS_2_/PLLA nanocomposites.

The thermal behavior of the different PLLA films and PLLA/HA/INT-WS_2_ nanocomposites films were measured using DSC. The results are summarized in Table 4, and the heating and cooling curves are presented in Figure 8. 

The addition of 40 wt% HA nanoparticles to the PLLA film increased the glass transition temperature (*T_g_*) (62.7 °C) by merely 1.9% compared to the neat PLLA film (61.5 °C). The addition of 0.5 wt% INT-WS_2_ to the PLLA film increased *T_g_* (66.7 °C) significantly by 8.5% compared to the *T_g_* of the neat PLLA film. Therefore, the PLLA film with 0.5 wt% INT-WS_2_ has the highest thermal deformation resistance of the films tested.

The cold crystallization temperature (*T_cc_*) of the PLLA film with 0.5 wt% INT-WS_2_ (107.9 °C) is lower than the *T_cc_* of the neat PLLA film (114.1 °C). In addition, the PLLA film with 40 wt% HA has lower *T_cc_* (93.6 °C) than both the neat PLLA film and the PLLA film with 0.5 wt% INT-WS_2_, which indicates that the PLLA with 40 wt% HA NPs film consists of smaller crystallites, compared to the neat PLLA film. The lower ΔH_cc_ of PLLA film with 40 wt% HA and PLLA film with 0.5 wt% INT-WS_2_ compared to the neat PLLA film also indicates the presence of bigger PLLA crystallites in the neat PLLA film, which is consistent with the findings from XRD (see discussion of Table 1 data, above).

The addition of 40 wt% HA nanoparticles to the PLLA reduced *T_m_* (177.6 °C) compared to the neat PLLA film (179.6 °C), while the addition of 0.5 wt% INT-WS_2_ resulted in increased *T_m_* (181.7 °C). Therefore, the PLLA film with 0.5 wt% INT-WS_2_ has the highest thermal stability [36]. The Δ*H_m_* values of the PLLA films with 40 wt% HA (33.2 J/g) and PLLA film with 0.5 wt% INT-WS_2_ (34.3 J/g) are lower compared to the PLLA film (39.1 J/g). Therefore, the HA nanoparticles and the INT-WS_2_ each, independently lower the energy required for breaking the polymer chain-chain interactions. The lower *T_c_* and higher Δ*H_c_* of PLLA film with 40 wt% HA (96.9 °C, 5.6 J/g) compared to the PLLA film (101.6 °C, 2.0 J/g), shows that the PLLA with 40 wt% HA film has a higher cooling rate [53,54,55] and smaller crystal nuclei. The higher *T_c_* and higher Δ*H_c_* of PLLA film with 0.5 wt% INT-WS_2_ (116.9 °C, 34.9 J/g) compared to the PLLA film, indicate that the PLLA film with 0.5 wt% INT-WS_2_ has a lower cooling rate and even smaller crystal nuclei. 

The degree of crystallinity (*X_c_* and (1 − *λ*)*_c_*) of the PLLA film with 40 wt% HA (32.2%, 6.0%) is higher compared to the neat PLLA film (7.5%, 2.2%), which indicates that the PLLA film with 40 wt% HA is harder and denser than the neat PLLA film. However, the *X_c_* and (1 − *λ*)*_c_* of PLLA film with 0.5 wt% INT-WS_2_ (33.5%, 36.7%) are even higher compared to the PLLA film with 40 wt% HA, therefore, the PLLA film with 0.5 wt% INT-WS_2_ is the hardest, and the densest film among the three [56,57,58]. However, the results of the micro-hardness test and the nanoindentation tests, show that the hardest film among the three is not the PLLA film with 0.5 wt% INT-WS_2_, but the PLLA film with 40 wt% HA. The reason for the difference between the estimated hardness trend and the mechanical measurements could possibly be linked to the nuclei size. The crystallites of the PLLA film with 0.5 wt% INT-WS_2_ are larger than the crystallites of the PLLA film with 40 wt% HA. Although this is a very different material, these results are consistent with the Hall–Petch effect, usually associated with polycrystalline metallic films. According to this law, as the size of the crystallites is reduced, the area of their grain boundaries increases; thereby increasing the hardness of the material [59,60].

PLLA films with 40 wt% HA and 0.25–0.75 wt% INT-WS_2_ have thermal properties (*T_g_*, *T_cc_*, *T_m_*, and *T_c_*) similar to the PLLA film with 40 wt% HA. Consequently, the PLLA film with 40 wt% HA and 0.25–0.75 wt% INT-WS_2_ have smaller thermal deformation resistance, crystallite size, thermal stability, and lower cooling rate compared with the PLLA film with 0.5 wt% INT-WS_2_. However, the PLLA film with 40 wt% HA and 0.25–0.75 wt% INT-WS_2_ have better thermal deformation resistance, smaller crystallites, smaller thermal stability, and lower cooling rate compared to the neat PLLA film. 

The ΔH_cc_ of PLLA film with 40 wt% HA and 0.5–0.75 wt% INT-WS_2_ (2.5–2.4 J/g) is lower compared to the other samples, which is attributed to the smaller crystallites in the nanocomposite films, due to the combined addition of HA nanoparticles and INT-WS_2_ to the PLLA film. The PLLA film with 40 wt% HA and 0.25 wt% INT-WS_2_ has lower ΔH_cc_ (4.8 J/g) compared to the neat PLLA film and higher ΔH_cc_ compared to the rest of the samples. This data demonstrates that the addition of a small amount of INT-WS_2_ combined with 40 wt% HA produced smaller crystallites compared to the neat PLLA film. The ΔH_m_ of the PLLA film with 40 wt% HA and 0.25–0.75 wt% INT-WS_2_ is lower compared to the other samples, thus the combined addition of HA nanoparticles and INT-WS_2_ to the PLLA film decreased the flexibility of the polymer chains and the energy required to break the interaction between the polymer chains.

PLLA films with 40 wt% HA and 0.25–0.75 wt% INT-WS_2_ have similar *X_c_* and (1 − *λ*)*_c_* to the PLLA film with 40 wt% HA, but lower *X_c_* and (1 − *λ*)*_c_* compared to the PLLA film with 0.5 wt% INT-WS_2_. The *X_c_* and (1 − *λ*)*_c_* of PLLA films with 40 wt% HA and 0.5 wt% and 0.75% INT-WS_2_ are very similar; thus they are equally hard. However, the PLLA film with 40 wt% HA and 0.25 wt% INT-WS_2_ has lower *X_c_* but higher (1 − *λ*)*_c_* compared to the PLLA film with 40 wt% HA and 0.5–0.75 wt% INT-WS_2_. Therefore, the PLLA film with 40 wt% HA and 0.25 wt% INT-WS_2_ is more elastic and but not as hard as the PLLA films with 40 wt% HA and 0.5–0.75 wt% INT-WS_2_. This is in agreement with the results of the mechanical properties (Section 3.3). 

### 3.7. Micro-Raman Spectroscopy

**Figure 9:**
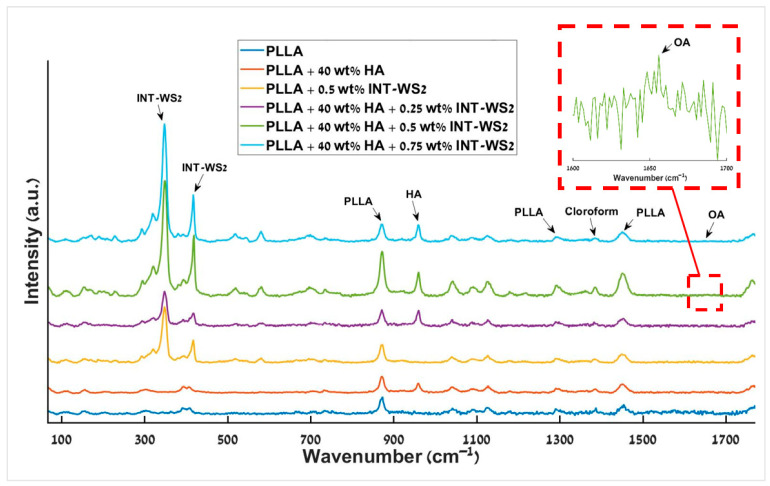
Raman spectra of the different PLLA films and PLLA/HA/INT-WS_2_ nanocomposites films. The Raman peak of the oleic acid is shown in the inset.

The Raman spectra of the different PLLA films and PLLA/HA/INT-WS_2_ nanocomposite films are presented in Figure 9. The PLLA film with 40 wt% HA and the neat PLLA film has exactly the same pattern of peaks as well as identical energies (873 cm^−1^, 1452 cm^−1^) [61,62], except for the peak of the HA at 960 cm^−1^ [63]. In addition, comparing the PLLA film with 0.5 wt% INT-WS_2_ to the neat PLLA film also shows the same pattern of peaks and intensity, except for the peaks of the INT-WS_2_ at 350 cm^−1^ and 418 cm^−1^ [64]. The match between the different spectral patterns is excellent, indicating that no chemical reaction took place between the different ingredients of the nanocomposite, as all the identified peaks belong to the pure reagents, with no missing peaks. Hence the chemical composition of the PLLA was not affected by the addition of the HA NPs and INT-WS_2_, or from the preparation of the film as suggested above (Section 3.1).

The band at 1379 cm^−1^ is associated with chloroform [65]. That band can be seen in the spectra of all the different PLLA films and PLLA/HA/INT-WS_2_ nanocomposites films. The existence of this peak indicates that residual amounts of the solvent remain in the films. The presence of solvent residues can significantly alter the mechanical properties of the nanocomposite film and its long-term behavior [66,67,68]. 

The film of PLLA with 0.5 wt% INT-WS_2_ and the films of PLLA with 40 wt% HA and 0.25–0.75 wt% INT-WS_2_ present peaks at 350 cm^−1^ and 418 cm^−1^, which are associated with the E_2g_ and A_1g_ modes of the INT-WS_2_ [69]. 

OA is a component that was incorporated only into the films of PLLA with 40 wt% HA and 0.25–0.75 wt% INT-WS_2_ (see Section 2.1). However, the main peak associated with the OA at 1655 cm^−1^ [70] is rather small and can be observed by focusing on the portion of the spectrum near this peak (red dashed square) and magnifying the scale. The low intensity of the peak reflects the fact that the OA concentration is very low in the films (150 µL). 

**Figure 10:**
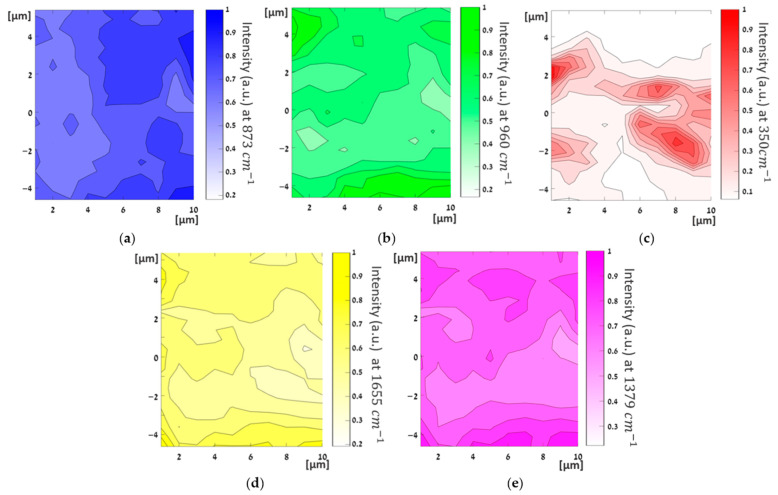
Raman intensity mapping of PLLA film with 40 wt% HA and 0.5 wt% INT-WS_2_ on an area of (10×10 µm^2^). Blue—PLLA at 873 cm^−1^ (**a**), green—HA at 960 cm^−1^ (**b**), red—INT-WS_2_ at 350 cm^−1^ (**c**), yellow—OA at 1655 cm^−1^ (**d**), and purple—chloroform at 1379 cm^−1^ (**e**).

Raman intensity mappings of the PLLA with 40 wt% HA and 0.5 wt% INT-WS_2_ films were carried out and are displayed in Figure 10.

Intensity mapping of the PLLA peak at 873 cm^−1^ (blue) shows a relatively uniform Raman light scattering intensity on the entire scanned area, with a minimum value of 60% with respect to the maximum (normalized) intensity. This indicates, as suggested above, that the PLLA film was uniform and that it was not affected by the addition of the solvent, HA, or and INT-WS_2_, nor from the fabrication process of the film. The result also shows that no chemical reactions occurred between the four main components during their mixing and processing of the film. Furthermore, the intensity mapping of HA NPs at 960 cm^−1^ (green) shows a good dispersion of the HA nanoparticles in the film, which confirms the observation of a uniform HA distribution obtained via SEM imaging (Section 3.2).

Notwithstanding the limited resolution of the technique (>1 μm), the INT-WS_2_ are clearly seen as elongated red shapes throughout the film in the Raman mapping (red features in Figure 10c). Obviously, the asymmetric shape of the nanotube does not reflect its genuine shape, since the coarse size of the focused laser beam (1–2 μm) is at least 10 times larger than the tube diameter (~100 nm). Moreover, it can be seen that the nanotubes are fully dispersed in the film. Their long axis seems to lie in the film plane with orientation roughly in the x-direction. The preferred directionality of the tubes could be related to the mode of evaporation of the solvent from the casted film. For example, a higher evaporation rate from the container wall could induce radial flow, which would orient the tubes accordingly with their long axis parallel to the (radial) flow direction. 

Raman mapping of OA at 1655 cm^−1^ (yellow) presents relatively strong and uniform intensity throughout the film area, with a minimum value of (normalized) intensity around 40%. Thus, it can be concluded that the OA was uniformly dispersed throughout the nanocomposite film during its preparation. Noticeably, the Raman mapping of the HA (green); OA (yellow), and chloroform (purple) exhibit some similarities. As for the OA, which is purposely used as the surfactant for the HA, the similar spatial distribution is not that astonishing. The analog spatial distribution of the chloroform and the HA indicates that the remnants of the solvent are adsorbed tightly to the HA surface and cannot be easily driven out, even after the long drying procedure used here.

The (normalized) intensity mapping of chloroform at 1379 cm^−1^ (purple) shows that it is uniform throughout the film. This indicates that notwithstanding the efforts to remove it, non-negligible amounts of chloroform residues (ca. 1 wt%) remained in the film, which correlates well with the observed feature in the Raman spectra above. Given the lengthy drying process under vacuum and heating at 45 °C, it is most likely that the residual chloroform was chemisorbed to one of the film ingredients and could not be easily removed from the film.

### 3.8. FTIR Spectroscopy

**Figure 11:**
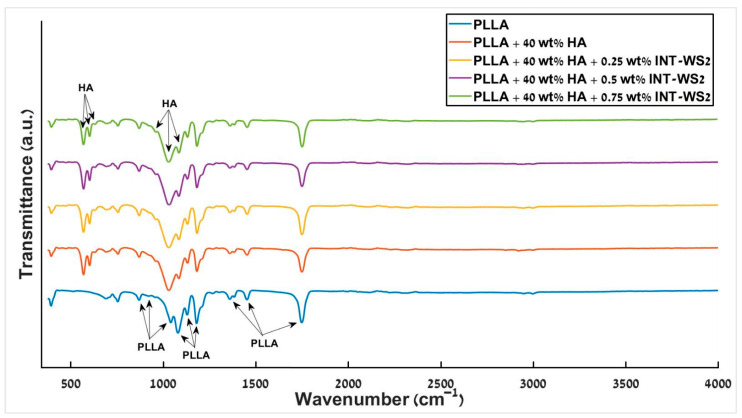
FTIR spectra of different PLLA/HA/INT-WS_2_ nanocomposite films.

FTIR of the different PLLA/HA/INT-WS_2_ nanocomposite films was conducted and the resulting spectra are displayed in Figure 11. 

Visibly, PLLA peaks are observed for all different films at the same position, accounting for the fact that the peaks at 1044 cm^−1^ and 1086 cm^−1^ [71] overlap with those of HA at 1033 cm^−1^ and 1093 cm^−1^ [72]. No extra peaks occur due to the addition of HA and nanotubes to the PLLA.

The nanotube peaks (<500 cm^−1^) [73] are not visible due to the dominant PLLA peak in this region. Thus, in agreement with the previous measurements, the FTIR results indicate that the four components (PLLA, HA, OA, and INT) are mixed together uniformly and are compatible with each other.

## 4. Discussion

The current work builds upon a previous work [36], where a nanocomposite of PLLA with small amounts of WS_2_ nanotubes dissolved in dichloromethane (DCM) was cast into a film. In this earlier work, the major obstacle was to fine-tune the drying procedure, which proved to be rather tricky. The PLLA-WS_2_ nanotube composites were dried in a vacuum oven at 30 °C for one week while monitoring the weight loss. This temperature was adopted after many trials because it is lower than the DCM boiling temperature (39.6 °C) which is important to ensure uniformity of the film. As anticipated, films that were not properly dried exhibited poor mechanical properties. The present three-component nanocomposite drying process presented a more challenging task. HA could not be properly dispersed in DCM. Therefore, chloroform (boiling point 61.15 °C) was used as a solvent and oleic acid as a compatibilizer between the HA phase and the other two components (PLLA and INT-WS_2_). The chloroform is less volatile than DCM and hence a higher drying temperature (45 °C) was selected for the casting. The presence of minute amounts of solvent adversely affected the nanotube-PLLA interaction and consequently the mechanical properties of the film. In the future, solvent-free processing of the film can be envisaged, like hot-pressing, or extrusion, and subsequent 3D printing [74]. 

The control of the interfacial interaction between the two majority phases, i.e., PLLA and HA and the minority phase—INT-WS_2_ has major implications on the mechanical stability of the nanocomposite subject to different stress and environmental conditions. The oleic acid, which has been used in the past for compatibilizing HA in different polymer phases, was found to be indispensable here. First, both oleic acid [75] and HA nanoparticles [76] are nontoxic and biocompatible. The FTIR, XRD, and Raman measurements reported here do not reveal any specific chemical bonding between any of the four components (PLLA, HA, OA, and INT) used in this work or new phase formation during the preparation of the film. It remains to be seen if other specific functionalization processes of the nanotube surface could further improve their interfacial interaction with the matrix and influence the mechanical behavior of the nanocomposite without sacrificing its biocompatibility. In the absence of a specific interaction between the nanotubes and the polymer-HA, their large surface area and aspect ratio (50–100) as well as their mechanical strength (10–22 GPa), large strain (10%) [77], nontoxic nature [21], and their facile dispersion make them ideally suited for reinforcing biodegradable polymers, even if added in minute amounts (~0.2 at%, ~0.6 wt%) [36,78,79]. Beyond this limit the nanotubes start agglomerating and their effectiveness in the polymer matrix is gradually impaired. PLLA is only one of a class of biodegradable polymers being intensively studied in order to improve the quality of medical care and make life more sustainable [80,81]. Obviously, more work, including in vivo experiments, is needed to determine the usefulness and biocompatibility of the WS_2_ nanotubes for potential future use in medical technologies.

## 5. Conclusions

PLLA films containing both hydroxyapatite and WS_2_ nanotubes were prepared via solvent casting process. While hydroxyapatite increases the hardness of the composite film, its tensile properties are compromised. The addition of the nontoxic nanotubes partially mitigates the reduced tensile properties of the film, providing biocompatible films with high hardness and tensile properties. XRD analysis and thermal measurements indicate that this is due to a reduction in crystal nuclei size. The addition of the nanotubes also improves the thermal stability of the films. Such nanocomposites could find numerous applications in medical technologies. 

## Figures and Tables

**Table 1:** The degree of crystallinity of PLLA film and HA/INT-WS_2_/PLLA nanocomposites.

Sample Type	Degree of Crystallinity (%)	Average Crystallite Size (Å)		
	WPF	Scherrer	WPF	
			PLLA	HA
PLLA film	32.8 ± 0.9	170	171 ± 2	–
PLLA film with 40 wt% HA	37.1 ± 3.0	130	128 ± 5	291 ± 18
PLLA film with 0.5 wt% INT-WS_2_	33.2 ± 1.6	165	162 ± 2	–
PLLA film with 40 wt% HA and 0.25 wt% INT-WS_2_	38.2 ± 2.5	145	145 ± 5	331 ± 20
PLLA film with 40 wt% HA and 0.5 wt% INT-WS_2_	42.7 ± 2.7	140	144 ± 4	353 ± 19
PLLA film with 40 wt% HA and 0.75 wt% INT-WS_2_	42.1 ± 2.2	150	145 ± 4	342 ± 16

**Table 2:** The mechanical properties of PLLA film and HA/INT-WS_2_/PLLA nanocomposites from tensile testing.

Sample Type	Young’s Modulus (GPa)	Yield Strength (MPa)	Strain at Failure (%)	Toughness (MPa)
PLLA film	1.55 ± 0.15	31.0 ± 2.4	2.7 ± 1.3	0.6 ± 0.2
PLLA film with 40 wt% HA	2.4 ± 0.1	26.7 ± 1.1	2.1 ± 0.1	0.3 ± 0.1
PLLA film with 0.5 wt% INT-WS_2_	2.25 ± 0.2	44.6 ± 4.65	6.8 ± 1.0	2.4 ± 0.5
PLLA film with 40 wt% HA and 0.25 wt% INT-WS_2_	2.7 ± 0.4	42.5 ± 5.8	7.3 ± 1.0	2.6 ± 0.3
PLLA film with 40 wt% HA and 0.5 wt% INT-WS_2_	3.8 ± 0.5	62.7 ± 1.2	3.2 ± 1.6	1.4 ± 0.7
PLLA film with 40 wt% HA and 0.75 wt% INT-WS_2_	2.7 ± 0.35	39.6 ± 4.9	5.8 ± 0.7	1.8 ± 0.25

**Table 3:** Parameters determined from the nanoindentation of PLLA film and HA/INT-WS_2_/PLLA nanocomposites.

Sample Type	Young’s Modulus (GPa)	Hardness (GPa)
PLLA film	3.3 ± 0.4	0.16 ± 0.05
PLLA film with 40 wt% HA	4.9 ± 0.7	0.24 ± 0.06
PLLA film with 0.5 wt% INT-WS_2_	3.4 ± 0.7	0.18 ± 0.08
PLLA film with 40 wt% HA and 0.25 wt% INT-WS_2_	5.6 ± 1.2	0.36 ± 0.15
PLLA film with 40 wt% HA and 0.5 wt% INT-WS_2_	4.6 ± 0.8	0.25 ± 0.08
PLLA film with 40 wt% HA and 0.75 wt% INT-WS_2_	4.3 ± 0.6	0.22 ± 0.07

**Table 4:** Thermal properties of PLLA film and HA/INT-WS_2_/PLLA nanocomposites.

Sample Type	*T_g_* (°C)	*T_cc_* (°C)	Δ*H_cc_* (J/g)	*T_m_* (°C)	Δ*H_m_* (J/g)	*T_c_* (°C)	Δ*H_c_* (J/g)	*X_c_* (%)	(1 − *λ*)*_c_* (%)
PLLA film	61.5	114.1	32.1	179.6	39.1	101.6	2.0	7.5	2.2
PLLA film with 40 wt% HA	62.7	93.6	3.2	177.6	33.2	96.9	5.6	32.2	6.0
PLLA film with 0.5 wt% INT-WS_2_	66.7	107.9	3.1	181.7	34.3	116.9	34.2	33.5	36.7
PLLA film with 40 wt% HA and 0.25 wt% INT-WS_2_	62.9	93.5	4.8	177.3	31.1	97.2	8.5	28.3	9.2
PLLA film with 40 wt% HA and 0.5 wt% INT-WS_2_	62.9	92.6	2.5	177.2	32.6	98.3	5.5	32.3	5.9
PLLA film with 40 wt% HA and 0.75 wt% INT-WS_2_	62.6	95.2	2.4	177.0	32.3	99.3	5.0	32.1	5.4

## Data Availability

Not applicable.
